# *Neoboutonia melleri* var *velutina* Prain: in vitro and in vivo hepatoprotective effects of the aqueous stem bark extract on acute hepatitis models

**DOI:** 10.1186/s12906-018-2091-2

**Published:** 2018-01-22

**Authors:** Anne Marie Endougou Effa, Emilie Gantier, Thierry Hennebelle, Vincent Roumy, Céline Rivière, Théophile Dimo, Pierre Kamtchouing, Pierre Desreumaux, Laurent Dubuquoy

**Affiliations:** 1 Université Lille Nord de France, F-59000 Lille, France; 2 Inserm U995, 4e étage Est, Faculté de Médecine – Pôle Recherche, Place Verdun, F-59045 Lille, France; 3 Laboratoire de Physiologie Animale, Faculté des Sciences, Université de Yaoundé I, BP, 812 Yaoundé, Cameroon; 4 Département de Biologie des Organismes Animaux, Faculté des Sciences, Université de Douala, BP, 24157 Douala, Cameroon; 5 Laboratoire de pharmacognosie, EA 4481(GRIIOT), Université de Lille 2, Faculté de Pharmacie, F-59006 Lille, France; 6 CHU Lille, Service des Maladies de l’Appareil Digestif et de la Nutrition, Hôpital Claude Huriez, F-59037 Lille, France

**Keywords:** *Neoboutonia melleri* var. *velutina* Prain, Acute hepatitis, Preventive treatment, Antioxidant

## Abstract

**Background:**

Hepatitis is a liver inflammation caused by different agents and remains a public health problem worldwide. Medicinal plants are an important source of new molecules being considered for treatment of this disease. Our work aims at evaluating the hepatoprotective properties of *Neoboutonia velutina,* a Cameroonian medicinal plant.

**Methods:**

The aqueous extract has been prepared using phytochemical methods. HepG2 cells were used to assess anti-inflammatory properties of the extract at different concentrations. Acute hepatitis models (Carbon tetrachloride and Concanavalin A) were performed in mice receiving or not receiving, different extract doses by gavage. Liver injury was assessed using histology, transaminases and pro-inflammatory markers. Extract antioxidant and radical scavenging capacities were evaluated.

**Results:**

The extract led to a significant decrease in pro-inflammatory cytokine expression in vitro and to a remarkable protection of mice from carbon tetrachloride-induced liver injury, as shown by a significant decrease in dose-dependent transaminases level. Upon extract treatment, inflammatory markers were significantly decreased and liver injuries were limited as well. In the Concanavalin A model, the extract displayed weak effects.

**Conclusions:**

Taking into account underlying mechanisms in both hepatitis models, we demonstrate the extract’s radical scavenging capacity. *Neoboutonia velutina* displays a potent hepatoprotective effect mediated through radical scavenging properties.

**Electronic supplementary material:**

The online version of this article (10.1186/s12906-018-2091-2) contains supplementary material, which is available to authorized users.

## Background

Hepatitis is a liver inflammation that can be acute or chronic and may be caused by several different agents: viruses, alcohol, drugs or poisons. Acute hepatitis does not necessarily require treatment but its extreme forms can be severe and even fatal as in cases of fulminant hepatitis and alcoholic hepatitis (AH) [1]. In contrast, chronic hepatitis needs therapeutic care since it can lead to advanced liver diseases such as fibrosis, cirrhosis and hepatocellular carcinoma.

Viral hepatitis are the most threatening forms of the disease worldwide - especially types B and C that lead to chronic disease in hundreds of millions of people and are collectively the most common cause of liver cirrhosis and cancer [2, 3]. Aside from viral hepatitis, and despite few registered clinical trials [4], alcoholic liver disease (ALD), including AH, is recorded as the second leading cause of advanced liver diseases in developed countries [1, 5].

In past decades, intensive research on hepatitis has led to current treatment methods that include vaccines, immunomodulators, interferons, nucleoside analogs and corticoids. More potent drugs for viral hepatitis are available. But, considering either the cost of current and future treatments [3] and current treatment side effects, drug resistance, potential drug-drug interactions and non-responder patients, those treatments are far from satisfactory to ensure support for all patients and easy global treatment access [3, 5, 6]. Moreover, the question of the management of hepatitis remains open since most new treatments target viral eradication rather than liver injury prevention.

A therapeutic alternative could be medicinal plants which are extensively used in developing countries. These approaches have gained popularity in developed countries and have served as sources of new molecules for several decades [7]. Unfortunately, despite their use over centuries, data about medicinal plants are not sufficient to meet the criteria needed to support worldwide use [8]. A deeper knowledge of medicinal plant efficacy, toxicity and mechanisms of action could enhance their traditional use and possibly reveal interesting approaches for the development of efficient low-cost therapies with fewer side effects and widespread tolerance.

Several medicinal plants are used in Cameroonian traditional medicine to treat liver diseases. *Neoboutonia velutina* Prain (Euphorbiaceae) is used to fight against worms, to treat abdominal pain, stomach aches and malaria [9]. Euphorbiaceae have been used around the world for their numerous properties but the *Neoboutonia* genus is poorly characterized [10]. Thus, though a Cameroonian traditional healer claimed the efficacy of *Neoboutonia velutina* against human hepatitis, no data are available concerning either its efficacy or its toxicity and mechanism.

The aim of this study is to characterize the hepatoprotective effect of *Neoboutonia velutina* and its possible mechanism of action.

## Methods

### Reagents

The following reagents were obtained from Sigma, France: Tumor Necrosis Factor alpha (TNFα), Interferon gamma (IFNγ), Carbon tetrachloride (CCl_4_), olive oil, Concanavalin A (ConA), ammonium molybdate, 1, 1-diphenyl-2-picrylhydrazyl (DPPH), sodium phosphate, Vitamin C and silymarin. Methylprednisolone (MP) was obtained from Pfizer, France.

### Plant material, aqueous extract preparation and fractionation

Locally called “Abenelanga”, the plant material was collected in October 2011 in Cameroon and identified as *Neoboutonia velutina* Prain (NV) in the Cameroonian National Herbarium using comparison to the sample N°6711 SRFcam previously deposited by Letouzey. *Neoboutonia melleri* (Müll. Arg.) Prain is then a *Neoboutonia velutina* Prain synonym. Otherwise, other authors described *Neoboutonia velutina* Prain as *Neoboutonia melleri* (Müll. Arg.) Prain variety, hence the plant name *Neoboutonia melleri* (Müll. Arg.) Prain var. *velutina* (Prain) Pax & K. Hoffm [11].

*Neoboutonia velutina* stem bark was shade dried and comminuted. The comminuted powder (100 g) was macerated in 1 L of distilled water (*w*/*v*) for 24 h. The macerate was filtrated after 24 h and concentrated under reduced pressure using a rotary vacuum evaporator. The concentrated extract was lyophilized to obtain the dry aqueous extract (NVH) 10.52 g. Drug extract ratio was 10:1.05 (*w*/w). The following fractionation method was implemented: dry extract 4 g was re-suspended in 20 mL of distilled water (w/v) and then impregnated in a Sephadex LH-20 column. The impregnated extract was eluted on a gradient (1 L × 3) with a solvent mixture of water: methanol (10:0, 7:3, 5:5, 3:7, 0:10 *v*/v) to obtain main aqueous fractions. The third fraction, called F3 (57 mg), was further fractionated by Medium Pressure Liquid Chromatography (MPLC) using the solvent mixture water: methanol (100:0, 95:5, 90:10, 85:15, 80:20, 0:100 v/v). Five compounds of interest have been obtained: F3_9–10 (1 mg); F3_12–13 (2.5 mg); F3_17–18 (2.09 mg); F3_38–41(2.5 mg) and F3_52–53 (8.2 mg).

### Qualitative phytochemical analysis and compound identification

To highlight supposed bioactive compounds in the extract sample (NVH), analytical analyses were conducted. Polyphenols, alkaloids, tannins, glycosides, saponins, lipids, sterols and polyterpens presence in NVH have been qualitatively assayed referring to Trease and Evans method [12] using ferric chloride (polyphenols and tannins), Dragendorff’s reagent (alkaloids), Fehling’s reagent (glycosides), the foam test (saponins), the brown paper test (lipids) and Lieberman-Buchard’s reaction (sterols and polyterpens). Otherwise, alkaloids, proanthocyanidins, polyphenols, flavonoids, and other compound presence have been assayed by thin-layer chromatography (TLC) using various reagents: Dragendorff’s reagent (alkaloids), dimethylamino-cinnamaldehyde (proanthocyanidins), ferric chloride (polyphenols), Neu’s reagent (flavonoids), sulfuric anisaldehyde and vanillin (other compounds). Briefly, for TLC analysis, 20 μl of NVH (50 mg/mL) were deposited on a silica plate which was eluted using the mobile phase water: methanol: acetic acid (12.5:12.5:1). The plate was then dried before being soaked for 5 s in the appropriate reagent.

In addition, NVH and its third fraction (F3) were analyzed by HPLC (high-performance liquid chromatography) using acetonitrile at varying percentages and time ranges (0, 0, 5, 5, 10, 20, 100% respectively during 1–10, 10–15, 15–20, 20–25, 25–35, 35–50, 50–60 min). NVH was further analyzed by HR-LC-MS (high resolution liquid chromatograph mass spectrometer) approach. NMR method has been used to analyze F3 sub fractions (F3_9–10; F3_12–13; F3_17–18; F3_38–41 and F3_52–53).

### In vitro total antioxidant capacity (TAC) assay

The Total Antioxidant Capacity (TAC) of the NVH extract sample (NVH) was assessed as reported by Prieto et al. [13] with slight modifications. Briefly, 0.1 mL of different NVH concentrations (25–2250 μg/mL) was mixed with 1 mL of the reagent solution (0.6 M sulfuric acid, 28 mM sodium phosphate and 4 mM ammonium molybdate). The mixture was incubated at 95 °C for 90 min, and then cooled down at room temperature. Absorbance was measured at 695 nm with Vitamin C and silymarin being used as reference. Results are expressed in absorbance.

### In vitro ferric reducing antioxidant power (FRAP) assay

The ferric reducing capacity of the extract sample (NVH) was determined as described by Benzie and Strain [14] with some modifications. Briefly, to 75 μL of the extract or standard at increasing concentrations (25, 50, 100, 200 and 400 μg/mL), was added 2 mL of a freshly prepared working FRAP reagent. The mixture was incubated at room temperature for 12 min. Absorbance was measured (593 nm) after the incubation time. Vitamin C and BHT (Butylated Hydroxytoluene) were used as standard. The working FRAP reagent was prepared as required by mixing 25 mL acetate buffer (300 mM; pH 3.6), 2.5 mL TPTZ (2,4,6-tripyridyl-*s*-triazine) solution (10 mM in HCL 400 mM) and 2.5 mL FeCl_3_.6H2O solution (10 mM). Results are expressed in absorbance.

### In vitro DPPH free radical scavenging assay

The radical scavenging capacity of the extract sample (NVH) was evaluated using the stable free radical DPPH assay [15] with slight modifications. Briefly, 50 μL of different NVH concentrations (25–2250 μg/mL) was incubated with or without 100 μL of an ethanolic solution of DPPH (50 μg/mL). Absorbance was measured (517 nm) every 5 min until 120 min. Vitamin C and silymarin were used as reference. DPPH results are expressed in percentage of inhibited DPPH.

The DPPH scavenging capacity of NVH and its main fractions was also assessed by TLC method [16] with some modifications using silica plates as the stationary phase and a water: methanol: acetic acid (12.5:12.5:1) mix as the mobile phase. Twenty μl of NVH (50 mg/mL) and each of the main fractions were deposited on a silica plate which was eluted and dried before being soaked for 5 s in a DPPH methanolic solution (2 mg/mL).

### Cell culture and in vitro anti-inflammatory assay

HepG2 cells were grown in an incubator (37 °C; 5% CO_2_) and maintained in supplemented DMEM (10% FBS; 1% antibiotics). For assay, cells (2 × 10^5^ per well), were stimulated with TNFα/IFNγ (50 ng/mL and 100 ng/mL respectively) then, treated 24 h later, with different NVH concentrations (1, 10 and 100 μg/mL). Dexamethasone (10^− 6^ M) was used as standard drug. Cells were harvested 24 h after treatment then frozen at − 80 °C for mRNA extraction and RT-qPCR analysis.

### Animals

Six-week-old C57BL/6 male mice (20–25 g) were purchased from JANVIER LABS and acclimated for at least one week before use. They were kept in a controlled environment (12 h light/dark cycles) and fed a standard rodent pellet diet ad libitum. Animal experiments were performed in accredited facilities (n° A59–35015) according to European governmental guidelines (n°2010/63/UE) and French national law (“Décret” n° 2013–118). The protocol received approval from both the local ethics committee of the University of Lille (France): “Comité d’Ethique en Expérimentation Animale Nord-Pas-de-Calais” (CEEA 75) and the French Ministry of Post-Graduate Education and Research (n°01757.01).

### In vivo CCl_4_-induced hepatitis

**Fig. 1:**
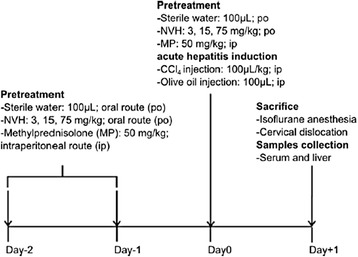
Diagram CCl_4_-model experimental protocol

In a single independent experiment, 60 mice were randomly divided into six groups: Control (CTR), CCl_4_, CCl_4_ + NVH 3, CCl_4_ + NVH 15, CCl_4_ + NVH 75, CCl_4_ + MP50. Each group was consisting of 10 mice (*n* = 10) and 5 animals per cage (two cages per group). For three consecutive days, mice in CTR and CCl_4_ groups were orally pretreated once a day with sterile water (NVH vehicle) while the other groups were pretreated once a day either orally with 3 increasing NVH doses (3, 15 or 75 mg/kg respectively in CCl_4_ + NVH 3, CCl_4_ + NVH 15, and CCl_4_ + NVH 75 groups) or intraperitoneally with methylprednisolone (MP) at 50 mg/kg in the CCl_4_ + MP50 group. MP was used as an anti-inflammatory reference drug (Fig. 1 Diagram).

The third day, acute liver injury was induced concomitantly with the last NVH or MP treatment by intraperitoneal injection of CCl_4_ (100 μl/kg) in olive oil. The CTR group received olive oil (100 μL) by intraperitoneal injection. Mice were sacrificed 24 h after CCl_4_ injection by cervical dislocation under isoflurane anesthesia. Mice sera and liver samples were collected (Fig. 1 Diagram). Transaminases levels, both alanine aminotransferase (ALT) and aspartate aminotransferase (AST), were measured in serum. Antioxidant activity, mRNA expression of cytochrome P450 2E1 and pro inflammatory genes were quantified in liver samples. Histology was conducted on liver tissues. Sixty animals/sixty were analyzed.

### In vivo Concanavalin-A induced hepatitis

**Fig. 2:**
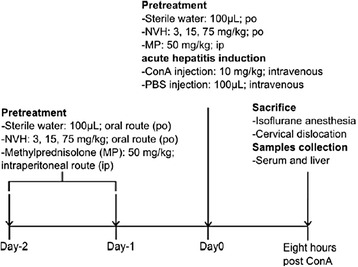
Diagram ConA-model experimental protocol

In two independent experiments, 80 mice were randomly divided into five groups of 10 mice or more (*n* ≥ 10): Control (CTR), ConA, ConA+NVH 15, ConA+NVH 75, ConA+MP50. For three consecutive days, mice in CTR and ConA groups were orally pretreated once a day with sterile water (NVH vehicle) while the other groups were pretreated once a day either orally with two NVH doses (15 or 75 mg/kg respectively in the ConA+NVH 15 and ConA+NVH 75 groups) or intraperitoneally with MP at 50 mg/kg in the ConA+MP50 group (Fig. 2 Diagram).

The third day, acute liver injury was induced concomitantly with the last NVH or MP treatment by intravenous injection of ConA (10 mg/kg) dissolved in PBS. The CTR group received PBS (100 μL) by intravenous injection. Eight hours after ConA injection, mice were sacrificed by cervical dislocation under isoflurane anesthesia. Mice sera and liver samples were collected (Fig. 2 Diagram). Transaminases levels, both ALT and AST, were measured in serum. Antioxidant activity and mRNA expression of pro inflammatory genes were quantified in liver samples. Histology was conducted on liver. Eighty animals/Eighty were analyzed. The extract dose of 3 mg/kg appeared not to be efficient in the CCl_4_ experiment then it was cancelled in ConA.

### Transaminases serum level

Blood samples were collected from isoflurane-anesthetized mice by retro-orbital puncture and centrifuged at 500 g for 10 min. Sera were collected and frozen at − 20 °C. ALT and AST serum levels were measured using an automatic analyzer (Hitashi 747 analyzer) and a clinical routine protocol according to manufacturer instructions [17].

### RT-qPCR quantified cytokines

Total mRNA was extracted from cells or liver pieces using a total mRNA isolation kit according to manufacturer protocol (Macherey-Nagel, Germany). Purified mRNA was quantified using a Nanodrop 1000 spectrophotometer. Complementary DNAs were obtained through reverse transcription using a high capacity cDNA reverse transcription kit according to the manufacturer protocol (Applied Biosystems, USA). Real-time qPCR was performed using the fast SYBR Green Master Mix (Applied Biosystems, USA) and the StepOnePlus Real-time PCR system (Applied Biosystems) at 25 °C using the following primers (forward and reverse respectively): human glyceraldehyde 3-phosphate dehydrogenase (GAPDH), GAG TCA ACG GAT TTG GTC GT and TTG ATT TTG GAG GGA TCT CG; human tumor necrosis factor alpha (TNFα), GGAGAAGGGTGACCGACTCA and CTGCCCAGACTCGGCAA; human interleukin 8 (IL8), AAGGAACCATCTCACTGTGTGTAAAC and AAATCAGGAAGGCTGCCAAGA; mouse GAPDH, ATG GGA AGC TTG TCA TCA ACG and GGC AGT GAT GGC ATG GAC TG; mouse TNFα, TGG GAG TAG ACA AGG TAC AAC CC and CAT CTT CTC AAA ATT CGA GTG ACA A; mouse interleukin 1 beta (IL-1β), CAACCAACAAGTGATATTCTCCATG and GATCCACACTCTCCAGCTGCA; mouse interleukin 6 (IL6), CTG ATG CTG GTG ACA ACC AC and TTC TGC AAG TGC ATC ATC GT; mouse cyclooxygenase 2 (COX-2), AGTTTGTTGAGTCATTCACCAGACA and CCACTGCTTGTACAGCAATTGG; mouse cytochrome P450 2E1, GTGACTGGGGAATGGGGAAA and AGGCTGGCCTTTGGTCTTTTT; mouse interferon gamma (IFNγ), GCTCTGAGACAATGAACGCT and AAAGAGATAATCTGGCTCTGC. Relative mRNA levels were calculated using the ∆CT method after normalization to GAPDH.

### Histology

Liver samples from different lobes were fixed overnight in phosphate-buffered formalin (4%). The samples were then embedded in paraffin, sectioned (4 μm thick slides) and stained with hematoxylin and eosin (H&E). Representative histopathological features (lesions or inflammatory cell infiltration), were examined by microscopy.

### Lipid peroxidation products in liver tissues

Lipid peroxidation was measured in mice liver tissues using a Thiobarbituric Acid-Reactive Substances (TBARS) assay kit according to manufacturer instructions (Cell Biolabs, France). To prevent lipid oxidation during the assay 100 mg liver tissue was homogenized in PBS (10%) containing 1X BHT. The homogenate was centrifuged at 10 g and 4 °C for 5 min. Then, 100 μL of the supernatant fraction was vigorously mixed with 100 μL of SDS lysis solution and incubated at room temperature for 5 min. After incubation, the thiobarbituric acid (TBA; 250 μL) was added to the mixture and incubation was carried out again, this time at 95 °C for 60 min. After the incubation time, samples were cooled for 5 min in an ice bath and then centrifuged at 3000 rpm for 15 min. Supernatants were collected. To prevent hemoglobin interference in samples, butanol (300 μL) was added to each unknown MDA supernatant (300 μL). The latter solution was vigorously mixed and centrifuged at 10 g for 5 min. Final butanol fractions were transferred to a microplate and absorbance was measured at 532 nm using a microtiter plate reader. TBARS concentrations were expressed as *n* moles of malondialdehyde (MDA) per milligram of tissue using an MDA standard.

### Glutathione content in liver tissues

The reduced glutathione (GSH) was measured in mice liver tissues according to Ellman’s method [18] using DTNB (2,2-dithio-5,5′-nitrodibenzoic acid). Indeed, 100 mg of liver tissue were homogenized (10%) in a Tris-HCl buffer (50 mM; pH 7.4). Homogenates were centrifuged at 10 g and 4 °C for 10 min. Supernatant fractions were collected for analysis. Twenty microliter of each supernatant was mixed with 20 μL of Tris-HCl buffer (50 mM; pH 7.4) and 3 mL of the Ellman’s reagent. The mixture was then incubated at room temperature for 60 min. The Ellman’s reagent was a solution of 4.96 mg DTNB in 250 mL phosphate buffer (0.1 M; pH 6.5). Absorbance was measured at 412 mM and final results were obtained using the molar extinction coefficient, ε _=_ 13,600/mol·cm.

### Superoxide dismutase activity in liver tissues

The total superoxide dismutase (SOD) was quantified in liver tissues according to Misra and Fridovich method [19] with slight modifications. Indeed, 134 μL of previously obtained liver homogenates was mixed with 1666 μL of carbonate buffer (0.05 M; pH 10.2). To initiate the reaction, 0.2 μL adrenalin (0.3 mM) was injected to the mixture using a microtiter plate reader. Absorbance was measured at 480 nm at the 20th and the 80th second after adrenalin injection. Protein concentration in samples was measured with BSA as standard using a Bradford reagent according to manufacturer instructions (Sigma, France). Results were expressed as unit of SOD per milligram of protein. One unit of SOD activity is defined as the amount of enzyme required to produce a 50% inhibition of adrenalin oxidation.

### Catalase activity in liver tissues

The catalase (CAT) activity was determined in mice liver tissues as described by Sinha [20] with slight modifications. Indeed, 50 μL of sample was mixed with 750 μL of a phosphate buffer (0.1 M; pH 7.5). The reaction was initiated by adding 200 μL of hydrogen peroxide (H_2_0_2_; 50 mM) to the mixture. The latter was then incubated at room temperature for 1 min. After the incubation time, the reaction was stopped by adding 2 mL of a 1.25% dichromate/acetic acid solution. Samples were then incubated at 100 °C for 10 min and cooled at room temperature. The dichromate/acetic acid solution was prepared by adding slowly 150 mL of acetic acid in 50 mL of an aqueous dichromate solution (5%). Hydrogen peroxide (H_2_0_2_) was used as standard. Absorbance was measured at 570 nm. Results were expressed as unit per mg of protein. One unit of catalase activity is defined as the amount of enzyme that decomposes 1 mmole H_2_O_2_/min/mg protein.

### Statistical analysis

Data are expressed as mean ± SEM. Statistical analyses were performed using GraphPad Prism 5 software. The Mann-Whitney test was used for two independent groups. To compare untreated and treated (with various extract doses) groups, the Kruskal Wallis test was used. The post hoc analysis was performed using the Dunn’s post-test. Statistical significant difference was defined by *p* < 0.05.

## Results

### NVH general phytochemical content

Phytochemical qualitative analysis performed on NVH has revealed saponin and glycoside presence. On the contrary, NVH appeared not to contain enough polyphenols, alkaloids, tannins, sterols, polyterpens and lipids (Table 1).

**Table 1:** NVH qualitative phytochemical profile

Qualitative analysis	Aqueous extract (NVH)	TLC analysis	Aqueous extract (NVH)
Polyphenols	–	Polyphenols	–
Alkaloids	–	Alkaloids	–
Tannins	–	Proanthocyanidins	–
Sterols and polyterpens	–	Flavonoids	–
Glycosides	+	Radical scavenging capacity	+
Saponins	+	/	/
Lipids	–	/	/

(+): presence; (−): absence; TLC: Thin Layer Chromatography

### HPLC profile and compound identification

**Fig. 3:**
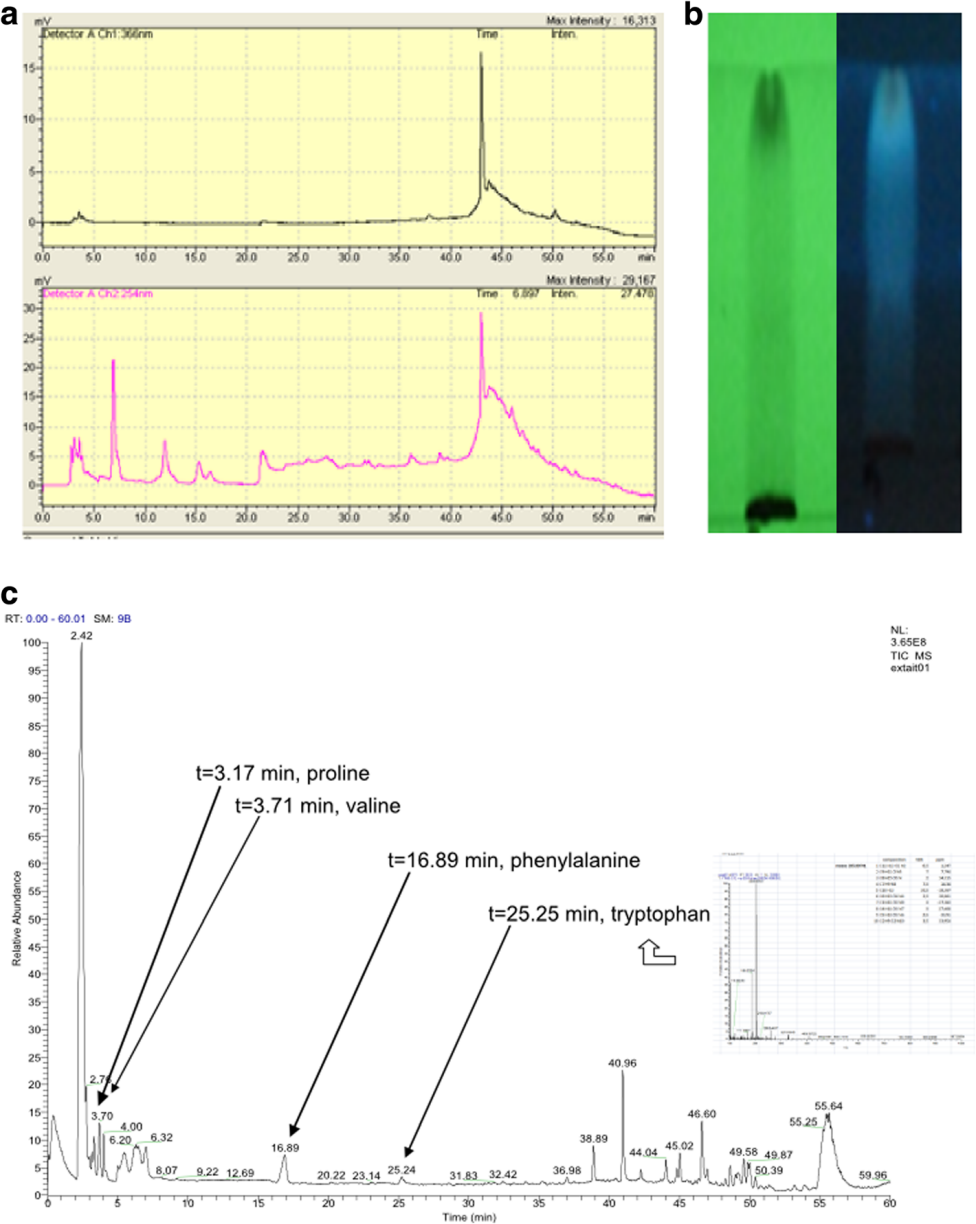
NVH HPLC, TLC and HR-LC-MS profiles. **a:** NVH HPLC profile using acetonitrile at different percentages and time ranges (0, 0, 5, 5, 10, 20, 100% respectively during 1–10, 10–15, 15–20, 20–25, 25–35, 35–50, 50–60 min). The upper part represents NVH HPLC profile at 366 nm and the lower part, NVH HPLC profile at 254 nm. **b**: NVH TLC fingerprint at 254 nm (left) and 366 nm (right) using water: methanol: acetic acid (12.5,12.5:1). **c**: NVH HR-LC-MS profile with the four identified amino acids

Looking at the HPLC profile, from the 40th to the 50th minute, main NVH compounds appeared as a large spike at 366 nm and 254 nm (Fig. 3a and b). With the HR-LC-MS approach, 12 significant mass pics were observed. Four could be attributed to known aminoacids (based on molecular formulas suggested in an acceptable range of homology with theoretical mass of these compounds): proline, valine, phenylalanine and tryptophan. Unidentified compounds correspond to molecular formulas that could neither be attributed to common proteogenic and non-proteogenic aminoacids, oligopeptides, purine and pyrimidine derivatives or common alkaloids – despite a high suggested number of nitrogen atoms – nor do the unidentified compounds hint at any relationship with a reasonable range of phytochemical groups previously encountered in our research (Fig. 3c).

**Fig. 4:**
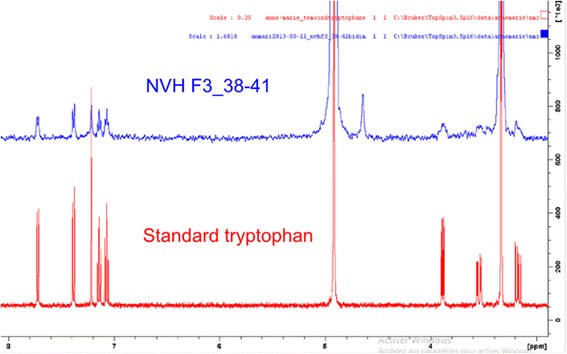
Tryptophan identification from F3 fraction. In blue: NVH F3_38–41, a compound isolated from F3 fraction; In red: standard tryptophan. The isolated compound from F3 fraction has been compared to tryptophan by NMR

On the other hand, the compound F3_38–41 obtained from F3 using fractionation has been identified by NMR as the amino acid tryptophan when compared with a standard tryptophan (Fig. 4). This isolated tryptophan from F3 represents 4.40% of the F3 fraction and 0.063% of the total extract NVH. The four other isolated compounds (F3_9–10; F3_12–13; F3_17–18; F3_52–53) remain to be identified.

### NVH displays in vitro anti-inflammatory properties

**Fig. 5:**
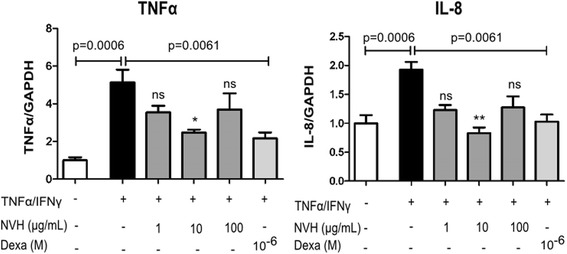
Pro-inflammatory cytokine expression in cells. Cells were stimulated or not (control) with TNFα/IFNγ, and then treated 24 h later with varying extract concentrations (1, 10 and 100 μg/mL) or with dexamethasone (10^− 6^ M). All experiments were performed in triplicate. Bar graphs represent cytokine expression in cells. The left panel represents TNFα and the right panel IL-8. Data are expressed as mean ± Standard Error of the Mean. Significant Dunn’s post tests are indicated as *p < 0.05; **p < 0.01; ***p < 0.001; ns: non-significant. The *p*-value indicates the Mann-Whitney test. NVH: *Neoboutonia velutina* aqueous extract; Dexa: Dexamethasone; TNFα: Tumor Necrosis Factor alpha; IFNγ: Interferon gamma; IL-8: Interleukin-8; GAPDH: Glyceraldehyde-3-Phosphate Dehydrogenase

HepG2 cells were cultured under inflammatory conditions to mimic liver inflammation encountered during hepatitis. Inflammatory cytokine expression (TNFα, IL-8) was then measured by RT-qPCR. As expected, HepG2 stimulation with TNFα and IFNγ led to a significant increase in TNFα (5 fold) and IL-8 (2 fold) expression when compared to CTR. In contrast, NVH treatment at 10 μg/mL resulted in significant decreases of TNFα (52%) and IL-8 (57%) levels (Fig. 5) when compared to TNFα/IFNγ group. The Kruskall Wallis *P* value summary (P) was 0.0496 (TNFα) and 0.0029 (IL-8). As expected, dexamethasone treatment at 10^− 6^ M led to a significant decrease in TNFα (52%) and IL-8 (42%) expression similarly to NVH.

### NVH demonstrates preventative properties against CCl_4_-induced liver injury

**Fig. 6:**
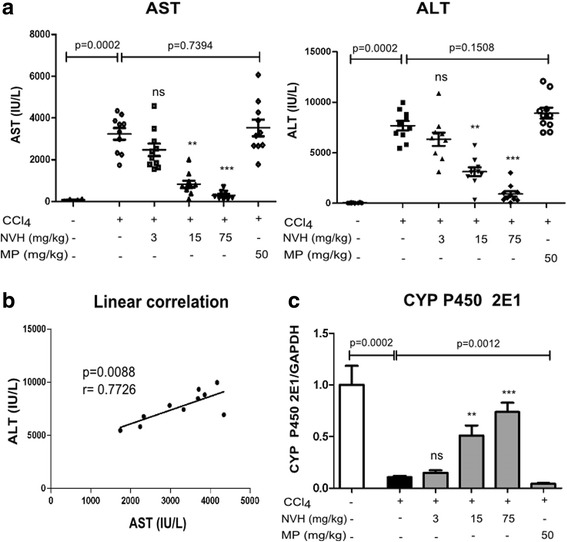
Transaminases serum level and CYP 2E1 expression in CCl_4_-intoxicated mice. Animals were pretreated with NVH extract or methylprednisolone during 3 consecutive days. On the third day, acute liver injury was induced with CCl_4_. Mice were sacrificed 24 h after CCl_4_ injection. **a**: Dot plot showing Aspartate aminotransferase (left panel) and Alanine aminotransferase (right panel) serum level for each animal in 6 different groups. **b**: Correlation between Aspartate aminotransferase and Alanine aminotransferase serum level. **c**: CYP 2E1 expression. Bar graphs represent CYP 2E1 expression. Medians are indicated by a horizontal line. Significant Dunn’s post tests are indicated as *p < 0.05; **p < 0.01; ***p < 0.001; ns: non-significant. The *p*-value indicates the Mann-Whitney test; experiment conducted once; *n* = 10 in each group. AST: Aspartate aminotransferase; ALT: Alanine aminotransferase; NVH: *Neoboutonia velutina* aqueous extract; MP: Methylprednisolone; CCl_4_: Carbon Tetrachloride

Compared to the CTR group, CCl_4_ injection induced significant increase of AST (40 fold) and ALT (213 fold) serum levels. While NVH treatment led to significant dose-dependent transaminases decrease reaching 91% (AST) and 88% (ALT) at the highest dose (75 mg/kg), compared to vehicle-treated CCl_4_ group. P value was < 0.0001 (Fig. 6a). A positive correlation has been observed between AST and ALT (Fig. 6b). In addition, CCl_4_ injection induced a significant decrease of cytochrome P450 2E1 (89%) when compared to the CTR group. NVH treatment on the contrary exhibited a significant dose-dependent re-establishment of cytochrome P450 2E1. When compared to CCl_4_ group, this is about a 1 fold, 5 fold and 7 fold increase respectively at 3 mg/kg, 15 mg/kg and 75 mg/kg (Fig. 6c). However, MP treatment did not prevent CCl_4_-induced damages on transaminases and cytochrome P450 2E1.

**Fig. 7:**
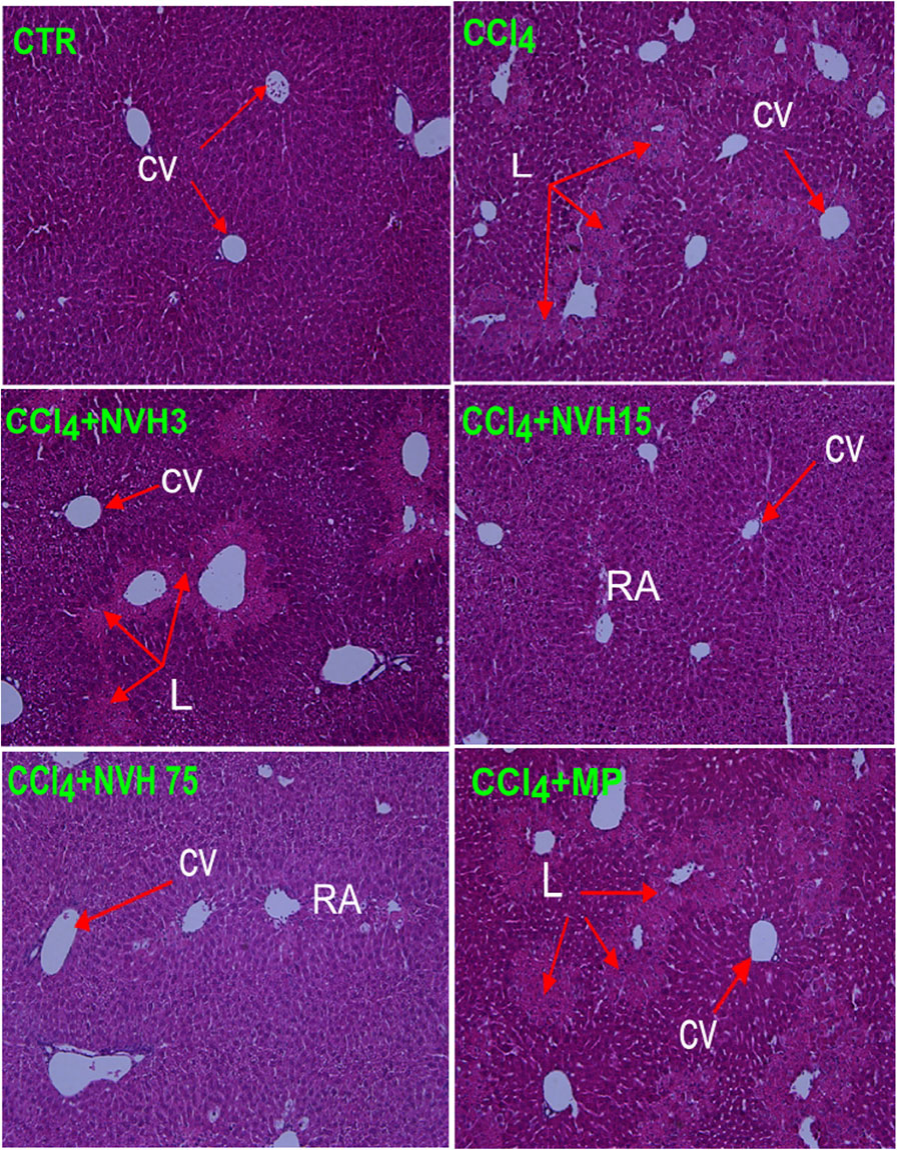
Histological changes in CCl_4_-intoxicated mice. Animals were pretreated with NVH extract or methylprednisolone during 3 consecutive days. On the third day, acute liver injury was induced with CCl_4_. Mice were sacrificed 24 h after CCl_4_ injection. Liver sections were fixed and paraffin-embedded before haematoxylin-eosin staining. CTR: Control mice with a normal liver structure; CCl_4_: carbon tetrachloride-treated mice with a damaged liver; CCl_4_ + NVH (3, 15 and 75): carbon tetrachloride and extract-treated mice with regenerating areas at highest doses; CCl_4_ + MP: carbon tetrachloride and methylprednisolone treated mice with persistent lesions. NVH: *Neoboutonia velutina* aqueous extract; MP: Methylprednisolone; CCl_4_: Carbon tetrachloride; CV: Centrilobular vein; L: Necrotic area; RA: Regenerating area. HEx100

Furthermore, liver injury was assessed using histological examination under microscope. When compared to CTR, vehicle-treated CCl_4_ mice displayed large and numerous lesions that were mostly located around centrilobular veins. In comparison, NVH-treated mice displayed few liver lesions with either smaller necrotic areas (15 mg/kg), necrotic areas under repair or an absence of necrotic areas (75 mg/kg). However, as observed with the lowest NVH dose (3 mg/kg), MP-treated mice displayed large, persistent lesions in accordance with their observed high transaminase levels (Fig. 7 and Table 2).

**Table 2:** Characteristic semi-quantitative evaluation of lesions

Groups	Lesions perimeter on 6.90 mm^2^ (mm)	Lesions number on 6.90 mm^2^
CTR	0.00 ± 0.00	00.00 ± 0.00
CCl_4_	1.05 ± 0.06 ^***^	12.20 ± 0.65 ^***^
CCl_4_ + NVH 3	0.96 ± 0.06	13.10 ± 0.66
CCl_4_ + NVH 15	0.78 ± 0.33 ^**£££**^	07.40 ± 1.48 ^**£££**^
CCl_4_ + NVH 75	0.35 ± 0.14 ^**£££**^	04.10 ± 1.30 ^**£££**^
CCl_4_ + MP	1.24 ± 0.12	10.90 ± 0.86

*p < 0.05; **p < 0.01; ***p < 0.001 compared to the CTR group; ^£^*p* < 0.05; ^££^*p* < 0.01; ^£££^*p* < 0.001 compared to CCl_4_-untreated mice

### NVH observed to limit CCl_4_-induced liver inflammation

**Fig. 8:**
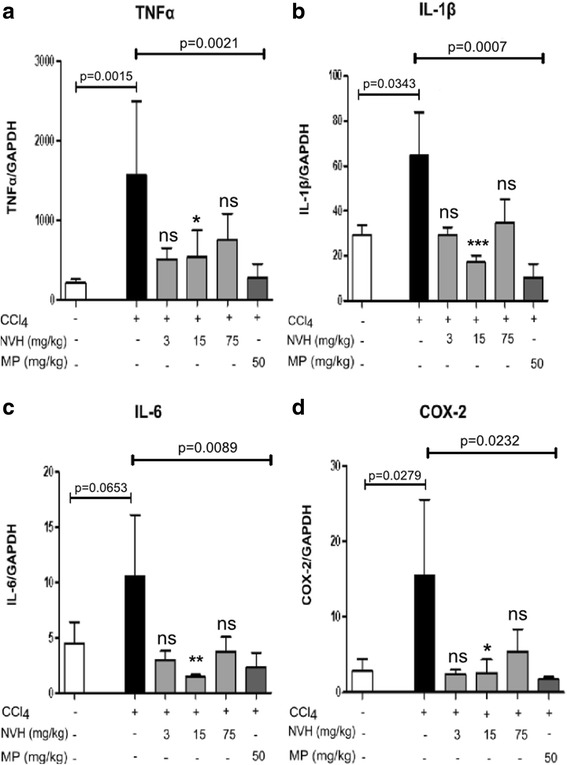
Pro-inflammatory mediator expression in CCl_4_-intoxicated mice. Mice were pretreated with the extract or methylprednisolone during 3 consecutive days. On the third day, acute liver injury was induced with carbon tetrachloride. Bar graphs present, Tumor Necrosis Factor alpha (**a**), Interleukin-1 beta (**b**), Interleukin-6 (**c**), and Cyclooxygenase-2 (**d**) liver expression in mice. Data are expressed as mean ± Standard Error of the Mean. Significant Dunn’s post tests are indicated as *p < 0.05; **p < 0.01; ***p < 0.001; ns: non-significant. The p-value indicates the Mann-Whitney test. One experiment conducted, n = 10 in each mice group. TNFα: Tumor Necrosis Factor alpha; IL1-β: Interleukin-1 beta; IL-6: Interleukin-6; COX-2: Cyclooxygenase-2; CCl_4_: Carbon tetrachloride; NVH: *Neoboutonia velutina* aqueous extract; MP: Methylprednisolone; GAPDH: Glyceraldehyde-3-Phosphate Dehydrogenase

Similarly, CCl_4_ injection significantly increased liver TNFα, IL-1β, IL-6 and COX-2 mRNA expression (7, 2, 2 and 6-fold respectively). These measurements were taken with RT-qPCR. Since its effect appears at 3 mg/kg, regardless of doses, NVH treatment prevents these CCl_4_-induced inflammatory markers from increasing (Fig. 8a-d). At 15 mg/kg, NVH displayed significantly decreased TNFα expression (66% compared to vehicle-treated CCl_4_ group). Similar significant effects were observed for IL-1β, IL-6 and COX-2 (73%, 86% and 83% decrease respectively) at 15 mg/kg (Fig. 8b-d). *P* values were 0.0022 (IL-1β), 0.0036 (IL-6, TNFα), 0.0461 (COX-2) at 15 mg/kg. MP exhibited the same profile with 83%, 84%, 78% and 89% decreases respectively in TNFα, IL-1β, IL-6 and COX-2 levels.

### NVH observed to prevent lipid peroxidation and deleterious effect of CCl_4_ on antioxidant

Malondialdehyde (MDA) has been quantified in CCl_4_-intoxicated-mice liver tissues to assess lipid peroxidation. Thus, CCl_4_ injection led to a non-significant increase of MDA (1.1 fold) compared to CTR. On the contrary, NVH pretreatment displayed a dose-dependent decrease of MDA level (18% at 15 mg/kg and 31% at 75 mg/kg) compared to CCl_4_ group. MDA decrease appeared significant at 75 mg/kg (*p* = 0.012). Similarly, MP exhibited a significant decrease of MDA (22%).

Furthermore, endogen antioxidants (GSH, SOD and CAT) have been assessed. Acute hepatitis induction led to a significant decrease of GSH content (21%) compared to CTR while NVH pretreatment prevents GSH lowering effect of CCl_4_. NVH protective effect appeared dose-dependent and significant at 15 and 75 mg/kg (*p* = 0.004). Similarly to NVH at 15 mg/kg, MP pretreatment significantly prevents GSH decrease.

In addition, CCl_4_ injection led to a significant increase of catalase (CAT; 3 fold) compared to CTR. NVH exhibited a non-significant dose dependent decrease of catalase compared to CCl_4_ group (12% decrease at 75 mg/kg). On the contrary, MP presented a significant decrease of catalase (48%).

**Fig. 9:**
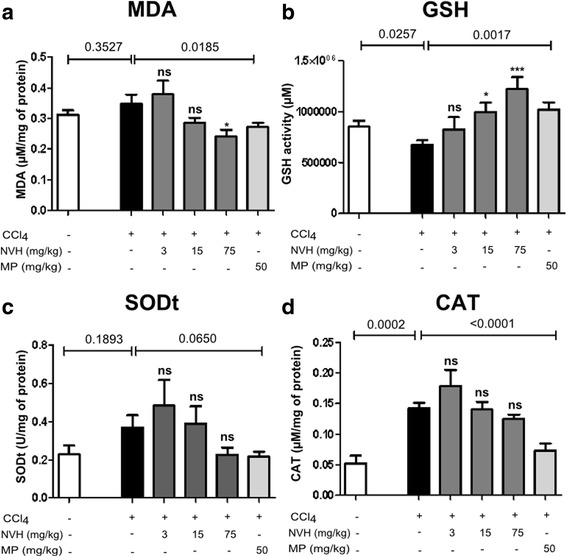
Lipid peroxidation product and endogen antioxidant activity in CCl_4_-intoxicated mice. Mice were pretreated with the extract or methylprednisolone during 3 consecutive days. On the third day, acute liver injury was induced with carbon tetrachloride. Bar graphs represent, Malondialdehyde level (**a**), Glutathione content (**b**), Total superoxide dismutase (**c**) and Catalase (**d**) activity in mice. Data are expressed as mean ± Standard Error of the Mean. Significant Dunn’s post tests are indicated as *p < 0.05; **p < 0.01; ***p < 0.001; ns: non-significant. The p-value indicates the Mann-Whitney test. One experiment conducted, n = 10 in each group. MDA: Malondialdehyde; GSH: Glutathione; SOD: Superoxide dismutase; CAT: Catalase; MP: Methylprednisolone

No significant differences have been noticed in SOD activity. Even though variations were non-significant, CCl_4_ injection led to an increase of SOD compared to CTR while NVH pretreatment exhibited a trend for a dose-dependent decrease. MP pretreatment displayed a non-significant decrease of SOD compared to CCl_4_ group (Fig. 9).

### NVH not seen to significantly prevent ConA-induced liver injury

**Fig. 10:**
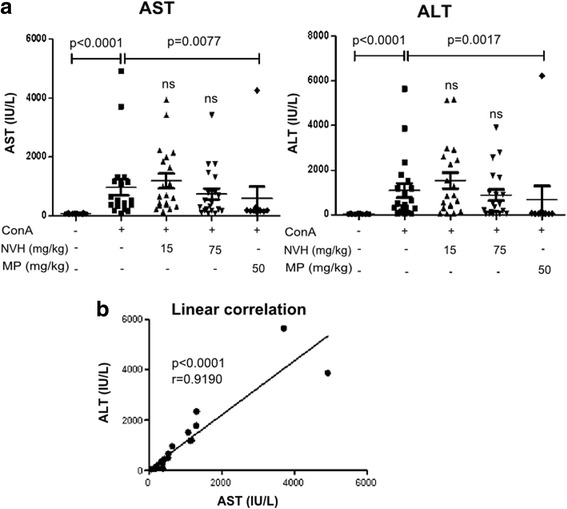
Transaminases serum level in Concanavalin A-induced liver injury in mice. Animals were pretreated with either NVH extract or methylprednisolone during 3 consecutive days and acute liver injury was immediately induced with Concanavalin A intravenous injection after the last treatment. All animals were sacrificed 8 h after Concanavalin A injection. **a**: Dot plot showing Aspartate aminotransferase (left panel) and Alanine aminotransferase (right panel) serum level for each mouse in 5 different groups. Medians are indicated by a horizontal line. Significant Dunn’s post tests are indicated as *p < 0.05; **p < 0.01; ***p < 0.001; ns: non-significant. The p-value indicates the Mann-Whitney test. Two independent experiments were conducted; *n* ≥ 10 in each group. **b**: Correlation between Aspartate aminotransferase and Alanine aminotransferase level. AST: Aspartate aminotransferase; ALT: Alanine aminotransferase; NVH: *Neoboutonia velutina* aqueous extract; MP: Methylprednisolone, ConA: Concanavalin A

**Fig. 11:**
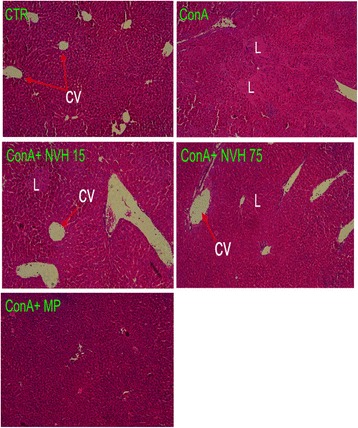
Histological changes in Concanavalin A-intoxicated mice. Animals were pretreated with either NVH extract or methylprednisolone during 3 consecutive days and acute liver injury was immediately induced with Concanavalin A intravenous injection after the last treatment. All animals were sacrificed 8 h after Concanavalin A injection. Liver sections were fixed and paraffin-embedded before haematoxylin-eosin staining. CTR: Control mice with normal liver structure; ConA: Concanavalin A treated mice with a damaged liver; ConA+NVH (15 and 75): Concanavalin A and extract treated mice with some persistent necrotic areas; ConA+MP: Concanavalin A and methylprednisolone treated mice without lesions. NVH: *Neoboutonia velutina* aqueous extract; MP: Methylprednisolone; ConA: Concanavalin A; CV: centrilobular vein; L: necrotic area. HEx100

As observed with CCl_4_, ConA injection led to a significant increase in AST (14 fold) and ALT (29 fold) 8 h after ConA injection. However, a non-significant slight decrease of AST (23%) and ALT (19%) (Fig. 10a) was observed upon NVH (75 mg/kg) treatment compared to vehicle-treated ConA group. In contrast, MP treatment displayed significant AST (39%) and ALT (38%) decrease. Both transaminases were correlated (Fig. 10b). Compared to CTR, vehicle-treated ConA mice displayed liver lesions located either in the tissue or around centrilobular veins. NVH treatment at 15 mg/kg did not prevent ConA induced-lesions while 75 mg/kg showed fewer lesions. In line with transaminases, MP pretreatment seemed to protect mice, as we did not notice lesions in MP treated-mice (Fig. 11).

### NVH did not significantly control ConA-induced inflammation

ConA intravenous injection significantly gave rise to TNFα (117 fold), IL-1β (9 fold), IL-6 (17 fold) and IFNγ (289 fold) mRNA expression measured by RT-qPCR in mice livers. NVH treatment did not significantly prevent the increase of pro-inflammatory cytokines since we noticed either a minimal or non-existent decrease compared to vehicle-treated ConA mice (Additional file 1: Figure S1A-D). Thus, NVH treatment led to 20% TNFα decrease (75 mg/kg), 20% IL-1β decrease (15 mg/kg) but neither IL-6 nor IFNγ decrease. Conversely, as expected, MP treatment significantly lowered TNFα (78%), IL-1β (76%), IL-6 (54%) and IFNγ (71%) expression (Additional file 1: Figure S1A-D).

### NVH effects on lipid peroxidation and antioxidant activity during ConA induced-hepatitis

MDA level and endogen antioxidant activity have been assayed in ConA-treated mice. Thus, acute hepatitis induction with ConA led to a significant increase in MDA level (1.9 fold), GSH content (2 fold) and catalase activity (3 fold) compared to CTR. On the contrary, NVH pretreatment resulted in a significant decrease in MDA level (*p* = 0.02; 37%) at 75 mg/kg and to a non-significant decrease of GSH and catalase at 75 mg/kg compared to ConA untreated group. Regardless the conditions, no significant variations of the total SOD were observed in the different groups. However, MP pretreatment resulted in a significant decrease in MDA level (36%), GSH content (46%) and catalase activity (52%) when compared to ConA untreated group (Additional file 2: Figure S2).

### NVH displays a weak total antioxidant capacity (TAC)

**Fig. 12:**
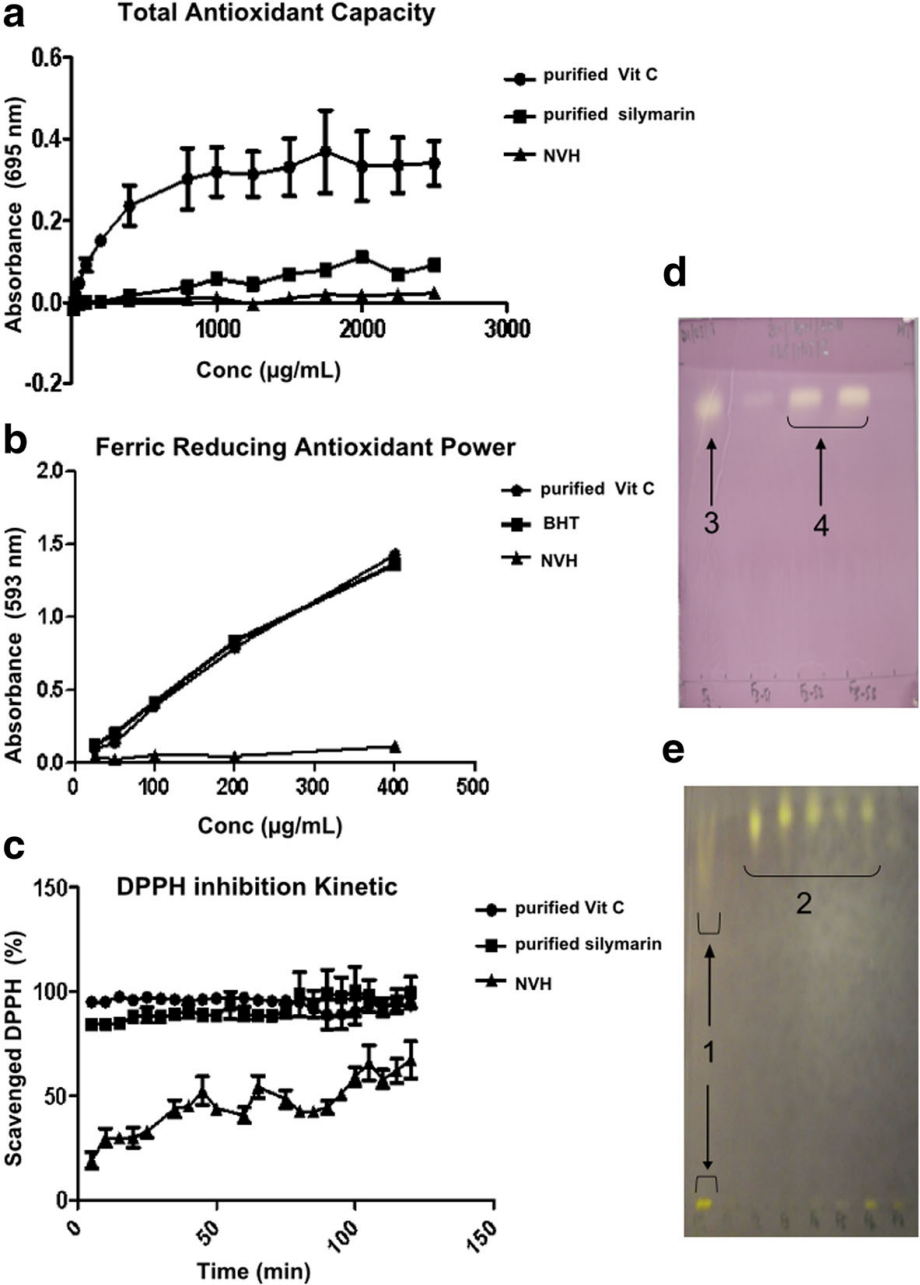
In vitro extract antioxidant capacity and ferric reducing antioxidant power (FRAP). **a**: graph showing total antioxidant capacity of the extract (triangle) compared to vitamin C (circle) and silymarin (square). Results are expressed as optical density mean ± Standard Error of the Mean. **b**: graph showing the ferric reducing capacity of the extract (triangle) compared to vitamin C (circle) and BHT (square). Results are expressed as optical density mean. **c**: DPPH scavenging capacity of the extract. In 3 different experiments, the extract (triangle) DPPH inhibition kinetic was assay within 120 min. Vitamin C (circle) and silymarin (square) were used as references. (**d**-**e**): DPPH inhibition by TLC method. The DPPH scavenging capacities of the extract and its main fractions were visualized on silica plates after elution in water: methanol: acetic acid (12.5/12.5/1). Yellow points represent the scavenging capacity of the extract and its main fractions and compound. One and 2 respectively represent the contact points of the extract and its main fractions with DPPH while 3 and 4 respectively represent the contact points of F3 fraction and F3_52–53 compound with DPPH. DPPH: 1,1-diphenyl-2-picrylhydrazyl; NVH: *Neoboutonia velutina* aqueous extract; TLC: thin layer chromatography; BHT: Butylated Hydroxytoluene

NHV prevents CCl_4_ but not ConA-induced liver injury. Oxidative stress is involved in the CCl_4_ mechanism. To understand the underlying mechanisms of NVH hepatoprotective action, we explored the direct antioxidant capacity of the extract sample in comparison with 2 well-known antioxidants: vitamin C and silymarin. Thus, a dose-dependent increase of the antioxidant capacity of vitamin C, silymarin and NVH were observed. Vitamin C capacity appeared highly marked reaching a plateau at the highest doses while silymarin and NVH capacity appeared continuous and slowly increasing (Fig. 12a).

### NVH presents a weak ferric reducing antioxidant power

The ferric reducing power of NVH has been assessed in vitro compared to vitamin C and BHT (Butylated Hydroxytoluene). NVH presents a very weak dose-dependent ferric reducing capacity while vitamin C and BHT on the contrary show a high dose dependent ferric reducing power. Vitamin C and BHT capacities appeared similar to each other (Fig. 12b).

### NVH displays a DPPH scavenging capacity

DPPH is a stable free radical widely used to estimate the scavenging capacity of antioxidants. In contact with an antioxidant, DPPH, normally blue, turns yellow. Thus, DPPH scavenging capacity of NVH was evaluated compared to that of silymarin and vitamin C. NVH exhibited a dose-time-dependent increasing scavenging capacity, reaching 65% inhibition after 120 min at its highest doses (2.25 mg/mL). As expected, vitamin C and silymarin appeared potent, reaching 95% inhibition within 5 min and then a plateau (Fig. 12c). Vitamin C and silymarin, which are purified compounds, appeared to be rapid, potent and constant radical scavengers while NVH, a non-purified compound mixture, seemed to be less rapid yet potent and constant.

Aiming at purifying the active compound, NVH fractionation was performed and led to antioxidant fraction identification by TLC. We then observed a diffuse yellow staining reflecting NVH and NVH fractions (NVHF) positive DPPH scavenging capacities. In comparison with NVH, the unpurified extract, NVHF displayed stronger DPPH scavenging capacity (Fig. 12e). In addition, the F3_52–53 compound obtained from F3 was positive to the DPPH assay (Fig. 12d). This antioxidant compound F3_52–53 represents 14.39% of the F3 fraction and 0.21% of the total extract NVH.

## Discussion

According to a Cameroonian traditional healer, NV (Euphorbiaceae) is a potent medicinal plant that acts against human forms of hepatitis. To our knowledge, this species has never been reported in ethnopharmacological studies and this new reported use is distinct from those previously described for related species of *Neoboutonia* [21, 22]. This genus has mostly been studied for non-polar compounds [23, 24], which are often unlikely to be found as significant components in traditional preparations involving water or hydroethanolic mixtures with rather low alcoholic amounts. It seemed promising to try and document this observation by characterizing NV hepatoprotective properties. We first investigated the aqueous extract (NVH) on HepG2 cells cultured under inflammatory conditions to mimic liver inflammation encountered during hepatitis. We observed decreased TNFα and IL-8 expression after NVH treatment. Our findings indicate an NVH anti-inflammatory potential in line with anti-inflammatory properties reported in other Euphorbiaceae plant family members [25]. This anti-inflammatory effect appears to be significant only at 10 μg/mL suggesting an optimal efficient dose of NVH between 1 and 10 μg/mL. As the lowest dose (1 μg/mL) seemed too low to optimally protect cells and the upper dose (100 μg/mL) announced toxicity in cells. When compared to NVH at 10 μg/mL, dexamethasone, known for its efficacy in acute-on-chronic pre-liver failure [26], displayed a similar anti-inflammatory effect by reducing TNFα and IL-8 expression.

Furthermore, NVH has been investigated in mice in a CCl_4_ hepatotoxic model [27]. CCl_4_-induced hepatotoxicity is a commonly used model to assess the hepatoprotective activity of a drug. Liver injury induced by CCl_4_ takes place via CYP P450 2E1 activity and results from the bio activation of CCl_4_ to CCl_3_^•^, the highly reactive trichloromethyl radical, then, in the presence of oxygen, to CCl_3_OO^•^, the more destructive trichloromethylperoxy radical [28–31]. These free radicals initiate lipid peroxidation and lead to the activation of Kupffer cells accompanied by the production of pro-inflammatory mediators such as TNFα, IL-1β, IL-6 and COX-2 resulting in hepatocellular damage and tissue inflammation. The induced liver damage elevates liver marker enzymes and releases them into the blood [32]. In our study, CCl_4_ injection led to a lowering of CYP 2E1 and gave rise to transaminases, TNFα, IL-1β, IL-6 and COX-2. An increase in transaminases level and pro-inflammatory markers have been reported by Tipoe and al. (2010) during CCl_4_-induced hepatitis [33] while Wong et al. (1998) reported a degradation of CYP 2E1 [34]. In contrast, NVH treatment led to significant improvement of those markers by reducing transaminases and regulating CYP 2E1. These results were associated with remarkable liver tissue repair under NVH treatment. Considering transaminases as hepatic injury biomarkers [35], our findings indicate a protective effect of NVH against hepatocyte destruction. These results concurred with the re-establishment of CYP 2E1 under NVH treatment, supporting that NVH could be a barrier against CCl_4_ bio activation via CYP 2E1 and thus against hepatocyte destruction. NVH therefore seems to act in CYP 2E1 regulation.

In line with limited TNFα and IL-8 levels in HepG2 cells treated with NVH, pro-inflammatory markers (TNFα, IL-1β, IL-6 and COX-2) decreased after NVH treatment in mice, which confirms NVH anti-inflammatory potential. This argues in favor of the protective effect of NVH against CCl_4_-induced liver damage.

To characterize NVH efficacy and its underlying mechanism, we investigated NVH in a ConA model which mimics autoimmune hepatitis. ConA-induced acute liver injury model is well established as a model of T cell-mediated liver injury [36–38]. Several cytokines are involved, including TNFα, IFNγ, IL-1β and IL-6. As reported in other studies [39], in our study, after ConA injection, we found increased transaminases levels in addition to up-regulated TNFα, IL-1β, IFNγ, IL-6 expression. In contrast to CCl_4_ model, NVH treatment led to a little change of inflammatory markers and transaminases. At studied concentrations, these results suggest a limited NVH effect in early phase ConA-induced inflammation.

Compared to MP, which is used for alcoholic hepatitis treatment [40], we noticed a remarkable NVH effect in the CCl_4_ model but not in the ConA model. On the contrary, MP displayed a protective effect in ConA model but not in CCl_4_ model. Considering CCl_4_ [29] and ConA [37, 41] discrepancies in hepatotoxic mechanisms, NVH mechanism is likely different from MP. While MP effect is mediated by immune response modulation [42], NVH mechanism might be mediated via CYP 2E1 regulation and the radical scavenging pathway.

To verify the latter assumption, we assessed NVH antioxidant capacity. We found in vitro, a subtle but continual total antioxidant capacity of the extract sample and an interesting dose and time dependent radical scavenging capacity for NVH and its fractions. In addition, a radical scavenging compound (F3_52–53) has been isolated. On liver samples, as expected, NVH treatment restored GSH content decreased by CCl_4_ effect. This result suggests an NVH capacity to regulate endogen antioxidant. Surprisingly, rather than a decrease in SODt and catalase activity after CCl_4_ injection – commonly reported during hepatitis [43, 44] – we observed an increased expression of those antioxidant enzymes whereas NVH treatment tended to lead to their improvement. This moderated NVH effect on endogen SODt and catalase under NVH treatment is consistent with its subtle and continual total antioxidant capacity in vitro. An explanation of these results could be the acute character of our study with the knowledge that NVH antioxidant potential increases with time. Likewise, little change in liver tissue SOD activity post-treatment for acute CCl_4_-induced hepatitis has been reported by Hu et al. (2008) [45].

Malondialdehyde (MDA) is known as a lipid peroxidation product in cells [46]. Thus, lipid peroxidation occurring after CCl_4_ bio activation can be evaluated by measuring MDA level in liver tissue. In our study, a decrease in MDA during NVH treatment was observed. This finding confirmed the protective effect of NVH against CCl_4_-induced lipid peroxidation.

In order to identify the supposed bioactive compounds, we explored the phytochemical composition of NVH. We found that NVH contains glycosides and saponins whereas polyphenols, alkaloids, tannins, flavonoids and lipids were absent. A radical scavenging compound, F3_52–53, was also isolated as well as tryptophan (F3_38–41). In addition, NVH exhibited a remarkably poor ferric-reducing antioxidant capacity. Considering that polyphenols exert their antioxidant capacity via two main mechanisms – hydrogen donor or as metal chelator [47, 48] – our latter result seems consistent with polyphenol absence.

Since it is known that the antioxidant potential of plants includes free radical scavenging, metal chelation and an increase in antioxidant enzymatic activity [49], the observed weak antioxidant and ferric-reducing capacity of NVH in contrast to its heightened radical scavenging capacity associated with the isolated radical scavenging compound (F3_52–53) might define NVH as mostly a radical scavenger. This result could explain its noteworthy effect in the CCl_4_ model. As a radical scavenger, the slight NVH effect in the ConA model is not so surprising because it has been demonstrated that cytokine reduction due to a free radical scavenger does not always accompany ConA hepatitis attenuation [41].

It is highly unlikely that any compound described in *Neoboutonia melleri* up to now [10, 50] could be responsible for the observed activity, supposing both species share constituents (which is possible, but not made certain by their taxonomical relationship). Our attempts at isolating one or several radical scavenging compounds responsible for NVH activity led to the isolation of the compound F3_52–53 but failed, however, to lead to its identification due to the low amount of extract and lack of possible comparison with known compounds or phytochemical classes through conventional spectroscopical means. However, tryptophan has been isolated by fractionation in NVH as compound F3_38–41 and identified by HR-LC-MS and NMR. As tryptophan has been reported to protect hepatocytes against reactive oxygen species-dependent cell death [51], NVH efficacy could be related to its tryptophan content. On the other hand, NVH hepatoprotective effect could be due to saponins known for their hepatoprotective [52], antioxidant and anti-inflammatory properties [53]. Thus, the global NVH efficacy could be due to a conjugated action of its tryptophan and saponins through CYP 2E1 regulation and its radical scavenging pathway. After all the assays conducted, while it may be unsatisfactory to not identify all the active compounds, these results certainly highlight presumed high activity and the chemical novelty of these trace compounds. These results also indicate an original aspect of NVH and offer a strong argument for the value of further research of its potential for human hepatitis treatment.

Several studies have reported hepatoprotective properties in medicinal plants. The advantage of this study is first and foremost the demonstrated capacity of the whole extract (not a purified compound) to remarkably provide continuous hepatoprotective properties at relatively low doses (15 mg/kg and 75 mg/kg) over the course of few administrations in an acute model. The doses used here generally correspond to those used for the purified compound [54]. This provides an interesting comparison to other whole plant extracts which demonstrate efficacy at higher doses (> 50 mg/kg) and after multiple administrations [45]. This aspect is important to ensure a safe use of NVH. Secondly, during our phytochemical assays on NVH, this plant appeared not to respond as commonly expected of medicinal plants. Polyphenols known to be potent antioxidants were absent, as well as flavonoids, tannins and alkaloids. Nevertheless, NVH efficacy (a whole extract, not a purified compound) appears equal to dexamethasone on cells and different from methylprednisolone in mice. Dexamethasone and methylprednisolone are both purified compounds. Thirdly, *Neoboutonia velutina* has never been studied before for its pharmacological properties. However, a traditional healer presented this plant as particularly potent in protection from liver injury with a single dose. This assumption appeared promising so we thought it important to test scientifically. The present study helps document this non-referenced traditional use of a plant which traditional healers claim for its particularly potent hepatoprotective effect after a single administration. Deep investigations could lead to the development of an affordable improved-traditional medicine (ITM).

## Conclusions

We have demonstrated for the first time a model-dependent NVH efficacy on acute toxic hepatitis. Our data are consistent with the traditional use of *Neoboutonia velutina* for human hepatitis treatment. This has been demonstrated by a remarkable hepatoprotective effect in a free-radical dependent model and by the absence of this effect in an immune-mediated model. Furthermore, NVH has been found to contain saponins, glycosides and tryptophan. Its hepatoprotective mechanism therefore, seems to be related to a conjugated action of these pharmacological classes through CYP 2E1 regulation and its radical scavenging pathway. Given these findings, *Neoboutonia velutina* appears to be an interesting target for the development of affordable and safe hepatitis treatment.

## Additional files

Additional file 1: **Figure S1.** Pro-inflammatory cytokine expression in Concanavalin A-intoxicated mice. Mice were pretreated with the extract or methylprednisolone and acute liver injury was induced with Concanavalin A intravenous injection after the last treatment. Bar graphs show Tumor Necrosis Factor alpha (A), Interleukin-1 beta (B), Interleukin-6 (C) and Interferon gamma (D) liver expression. Data are expressed as mean ± Standard Error of Mean. Significant Dunn’s post tests are indicated as *p < 0.05; **p < 0.01; ***p < 0.001; ns: non-significant. The *p*-value indicates the Mann-Whitney test. Two independent experiments; *n* ≥ 10 in each group. TNFα: Tumor Necrosis Factor alpha; IL-1β: Interleukin-1 beta; IL-6: Interleukin-6; IFNγ: Interferon gamma; NVH: *Neoboutonia velutina* aqueous extract; MP: Methylprednisolone; ConA: Concanavalin A; GAPDH: Glyceraldehyde-3-Phosphate Dehydrogenase. (TIFF 2650 kb)

Additional file 2: **Figure S2.** Lipid peroxidation product and endogen antioxidant activity in Concanavalin A-intoxicated mice. Mice were pretreated with the extract or methylprednisolone and acute liver injury was induced with Concanavalin A intravenous injection after the last treatment. Bar graphs show Malondialdehyde level (A), Glutathione (B), total Superoxide dismutase (C) and Catalase (D) activity in mice. Data are expressed as mean ± Standard Error of the Mean. Significant Dunn’s post tests are indicated as *p < 0.05; **p < 0.01; ***p < 0.001; ns: non-significant. The p-value indicates the Mann-Whitney test. Two independent experiments; n ≥ 10 in each group. MDA: Malondialdehyde, GSH: Glutathione; SOD: Superoxide dismutase; CAT: Catalase; NVH: *Neoboutonia velutina* aqueous extract; MP: Methylprednisolone (TIFF 1841 kb)

Additional file 3: **Figure S3.** NVH TLC for phytochemical analysis. Twenty μl of NVH (50 mg/mL) were deposited on a silica plate which was eluted using water: methanol: acetic acid (12.5:12.5:1). The eluted plate was dried before being soaked for 5 s in the appropriate reagent. A: sulfuric anisaldehyde reagent; B: sulfuric vanillin reagent; C: NEU reagent; D: FeCl_3_ reagent_;_ E: Dimethylamino-cinnamaldehyde (DMACA) reagent. (TIFF 923 kb)

Additional file 4: **File S4.** Raw data from the article. (XLS 569 kb)

## Abbreviations

AH: Alcoholic hepatitis
ALD: Alcoholic Liver Disease
ALT: Alanine aminotransferase
ANOVA: Analysis of Variance
AST: Aspartate aminotransferase
CCl_4_: Carbon tetrachloride
cDNA: complementary Deoxyribonucleic Acid
ConA: Concanavalin A
COX-2: Cyclooxygenase-2
CTR: Control
CV: Centrolobular vein
DMEM: Dulbecco’s Modified Eagle Medium
DPPH: 1,1-diphenyl-2-picrylhydrazyl
FBS: Fetal Bovine Serum
GAPDH: Glyceraldehyde-3-Phosphate Dehydrogenase
H&E: Hematoxylin and Eosin
HPLC: High Pressure Liquid Chromatography
IFNγ: Interferon Gamma
IL1-β: Interleukin-1 beta
IL-6: Interleukin-6
IL-8: Interleukin 8
L: Necrotic area
MP: Methylprednisolone
mRNA: Messenger Ribonucleic Acid
NV: *Neoboutonia velutina*
NVH: *Neoboutonia velutina* aqueous extract
NVHF: *Neoboutonia velutina* main aqueous fractions
PBS: Phosphate Buffered Saline
qPCR: quantitative Polymerase Chain Reaction
RA: Regenerating area
SEM: Standard Error of Mean
TAC: Total Antioxidant Capacity
TLC: Thin Layer Chromatography
TNFα: Tumor Necrosis Factor Alpha
WHO: World Health Organization

## References

[1] Spengler EK, Dunkelberg J, Schey R (2014). Alcoholic hepatitis: current management. Dig Dis Sci.

[2] WHO. Guidelines for the prevention, care and treatment of persons with chronic hepatitis B infection. Geneva: World Health Organization; 2015. 26225396

[3] WHO. Guidelines for the screening, care and treatment of persons with chronic hepatitis C infection. Geneva: World Health Organization; 2016. 27227200

[4] Shah VH (2010). Alcoholic liver disease: the buzz may be gone, but the hangover remains. Hepatology.

[5] Mathurin P, Lucey MR (2012). Management Of alcoholic hepatitis. J Hepatol.

[6] Pawlotsky J-M (2013). Hepatitis C virus: standard-of-care treatment. Adv Pharmacol San Diego Calif.

[7] Farnsworth NR, Akerele O, Bingel AS, Soejarto DD, Guo Z (1985). Medicinal plants in therapy. Bull World Health Organ.

[8] WHO. General guidelines for methodologies on research and evaluation of traditional medicine. Geneva: World Health Organization; 2000.

[9] Lemmens RHMJ, Louppe D, Oteng-Amoako AA. Plant resources of tropical africa 7(2). Timbers 2. PROTA Foundation.Wageningen, Netherlands/CTA, Wageningen, Netherlands 2012.

[10] Long C, Beck J, Cantagrel F (2012). Proteasome inhibitors from Neoboutonia melleri. J Nat Prod.

[11] Hutchinson J, Dalziel JM (1958). Flora of west tropical Africa.

[12] Trease GE, Evans WC (1983). Pharmacognosy.

[13] Prieto P, Pineda M, Aguilar M (1999). Spectrophotometric quantitation of antioxidant capacity through the formation of a phosphomolybdenum complex: specific application to the determination of vitamin E. Anal Biochem.

[14] Benzie IF, Strain JJ (1996). The ferric reducing ability of plasma (FRAP) as a measure of antioxidant power: the FRAP assay. Anal Biochem.

[15] Aquino R, Morelli S, Lauro MR, Abdo S, Saija A, Tomaino A (2001). Phenolic constituents and antioxidant activity of an extract of Anthurium Versicolor leaves. J Nat Prod.

[16] Cieśla Ł, Kowalska I, Oleszek W (2013). Free radical scavenging activities of polyphenolic compounds isolated from Medicago Sativa and Medicago Truncatula assessed by means of thin-layer chromatography DPPH˙ rapid test. Phytochem Anal PCA.

[17] Dharancy S, Body-Malapel M, Louvet A (2010). Neutrophil migration during liver injury is under nucleotide-binding oligomerization domain 1 control. Gastroenterology.

[18] Tissue EGL (1959). Sulfhydryl groups. Arch Biochem Biophys.

[19] Misra HP, Fridovich I (1972). The role of superoxide anion in the autoxidation of epinephrine and a simple assay for superoxide dismutase. J Biol Chem.

[20] Sinha AK (1972). Colorimetric Assay of catalase. Anal Biochem.

[21] Chifundera K (2001). Contribution to the inventory of medicinal plants from the Bushi area, south Kivu Province, Democratic Republic of Congo. Fitoterapia.

[22] Kuete V, Efferth T (2011). Pharmacogenomics of Cameroonian traditional herbal medicine for cancer therapy. J Ethnopharmacol.

[23] Namukobe J, Kiremire BT, Byamukama R (2014). Cycloartane triterpenes from the leaves of Neoboutonia macrocalyx L. Phytochemistry.

[24] Tene M, Tane P, de Dieu Tamokou J, Kuiate J-R, Connolly JD (2008). Degraded diterpenoids from the stem bark of Neoboutonia mannii. Phytochem Lett.

[25] Dimo T, Nguemfo EL, Nguelefack TB (2006). Antinociceptive and anti-inflammatory effects of the ethyl acetate stem bark extract of Bridelia scleroneura (Euphorbiaceae). Inflammopharmacology.

[26] Zhang X-Q, Jiang L, You J-P (2011). Efficacy of short-term dexamethasone therapy in acute-on-chronic pre-liver failure. Hepatol Res Off J Jpn Soc Hepatol.

[27] Ghaffari H, Venkataramana M, Nayaka SC (2013). Hepatoprotective action of Orthosiphon Diffusus (Benth.) methanol active fraction through antioxidant mechanisms: an in vivo and in vitro evaluation. J Ethnopharmacol.

[28] Goeptar AR, Scheerens H, Vermeulen NP (1995). Oxygen and xenobiotic reductase activities of cytochrome P450. Crit Rev Toxicol.

[29] Weber LWD, Boll M, Stampfl A (2003). Hepatotoxicity and mechanism of action of haloalkanes: carbon tetrachloride as a toxicological model. Crit Rev Toxicol.

[30] Akindele AJ, Ezenwanebe KO, Anunobi CC, Adeyemi OO (2010). Hepatoprotective and in vivo antioxidant effects of Byrsocarpus coccineus Schum. And Thonn. (Connaraceae). J Ethnopharmacol.

[31] Hamdy N, El-Demerdash E (2012). New therapeutic aspect for carvedilol: antifibrotic effects of carvedilol in chronic carbon tetrachloride-induced liver damage. Toxicol Appl Pharmacol.

[32] Huang G-J, Deng J-S, Chiu C-S, et al. Hispolon protects against acute liver damage in the rat by inhibiting lipid peroxidation, proinflammatory cytokine, and oxidative stress and downregulating the expressions of iNOS, COX-2, and MMP-9. Evid-Based Complement Altern Med 2012;2012:480714. 10.1155/2012/480714 PMC3195309 22013489

[33] Tipoe GL, Leung TM, Liong EC, Lau TYH, Fung ML, Nanji AA (2010). Epigallocatechin-3-gallate (EGCG) reduces liver inflammation, oxidative stress and fibrosis in carbon tetrachloride (CCl_4_)-induced liver injury in mice. Toxicology.

[34] Wong FW, Chan WY, Lee SS (1998). Resistance to carbon tetrachloride-induced hepatotoxicity in mice which lack CYP2E1 expression. Toxicol Appl Pharmacol.

[35] Clark JM, Brancati FL, Diehl AM (2003). The prevalence and etiology of elevated aminotransferase levels in the United States. Am J Gastroenterol.

[36] Tiegs G, Hentschel J, Wendel A (1992). A T cell-dependent experimental liver injury in mice inducible by concanavalin a. J Clin Invest.

[37] Schümann J, Prockl J, Kiemer AK, Vollmar AM, Bang R, Tiegs G (2003). Silibinin protects mice from T cell-dependent liver injury. J Hepatol.

[38] Al-Shamsi M, Shahin A, Mensah-Brown EP, Souid A-K. Derangements of liver tissue bioenergetics in Concanavalin A-induced hepatitis. BMC Gastroenterol. 2013;13(6) 10.1186/1471-230X-13-6 PMC3571906 23311450

[39] Liu D, Zhang X, Jiang L, Guo Y, Zheng C (2014). Epigallocatechin-3-gallate (EGCG) attenuates concanavalin A-induced hepatic injury in mice. Acta Histochem.

[40] Mathurin P, O’Grady J, Carithers RL (2011). Corticosteroids improve short-term survival in patients with severe alcoholic hepatitis: meta-analysis of individual patient data. Gut.

[41] Nakashima H, Kinoshita M, Nakashima M (2008). Superoxide produced by Kupffer cells is an essential effector in concanavalin A–induced hepatitis in mice. Hepatology.

[42] Sougioultzis S, Dalakas E, Hayes PC, Plevris JN (2005). Alcoholic hepatitis: from pathogenesis to treatment. Curr Med Res Opin.

[43] Colak E, Ustuner MC, Tekin N, et al. The hepatocurative effects of Cynara Scolymus L. leaf extract on carbon tetrachloride-induced oxidative stress and hepatic injury in rats. SpringerPlus. 2016;5(216) 10.1186/s40064-016-1894-1 PMC4771653 27026910

[44] Younis T, Khan MR, Sajid M. Protective effects of Fraxinus xanthoxyloides (wall.) leaves against CCl_4_ induced hepatic toxicity in rat. BMC Complement Altern Med. 2016;16(407) 10.1186/s12906-016-1398-0 PMC5078913 27776508

[45] Hu X-P, Shin J-W, Wang J-H (2008). Antioxidative and hepatoprotective effect of CGX, an herbal medicine, against toxic acute injury in mice. J Ethnopharmacol.

[46] Suhail M, Suhail S, Gupta BK, Bharat V (2009). Malondialdehyde and Antioxidant enzymes in maternal and cord blood, and their correlation in normotensive and preeclamptic women. J Clin Med Res.

[47] Lafka T-I, Lazou AE, Sinanoglou VJ, Lazos ES (2011). Phenolic and antioxidant potential of olive oil mill wastes. Food Chem.

[48] Yang J, Ou B, Wise ML, Chu Y (2014). In vitro total antioxidant capacity and anti-inflammatory activity of three common oat-derived avenanthramides. Food Chem.

[49] Khan RA, Khan MR, Ahmed M, et al. Hepatoprotection with a chloroform extract of Launaea Procumbens against CCl_4_-induced injuries in rats. BMC Complement Altern Med. 2012;12(114) 10.1186/1472-6882-12-114 PMC3492157 22862950

[50] Zhao W, Wolfender J-L, Mavi S, Hostettmann K (1998). Diterpenes and sterols from Neoboutonia melleri. Phytochemistry.

[51] Kimura T, Watanabe Y (2016). Tryptophan protects hepatocytes against reactive oxygen species-dependent cell death via multiple pathways including Nrf2-dependent gene induction. Amino Acids.

[52] Dong D, Zhang S, Yin L (2013). Protective effects of the total saponins from Rosa Laevigata Michx fruit against carbon tetrachloride-induced acute liver injury in mice. Food Chem Toxicol Int J Publ Br Ind Biol Res Assoc.

[53] Chen Y, Miao Y, Huang L (2014). Antioxidant activities of saponins extracted from radix Trichosanthis: an in vivo and in vitro evaluation. BMC Complement Altern Med.

[54] Lee KJ, Choi JH, Jeong HG (2007). Hepatoprotective and antioxidant effects of the coffee diterpenes kahweol and cafestol on carbon tetrachloride-induced liver damage in mice. Food Chem Toxicol Int J Publ Br Ind Biol Res Assoc..

