# Supplementary figures and images for: Neoboutonia melleri var velutina Prain: in vitro and in vivo hepatoprotective effects of the aqueous stem bark extract on acute hepatitis models
Source: BMC Complement Altern Med. 2018 Jan 22;18:24. doi: 10.1186/s12906-018-2091-2 (PMC5778785; doi:10.1186/s12906-018-2091-2)

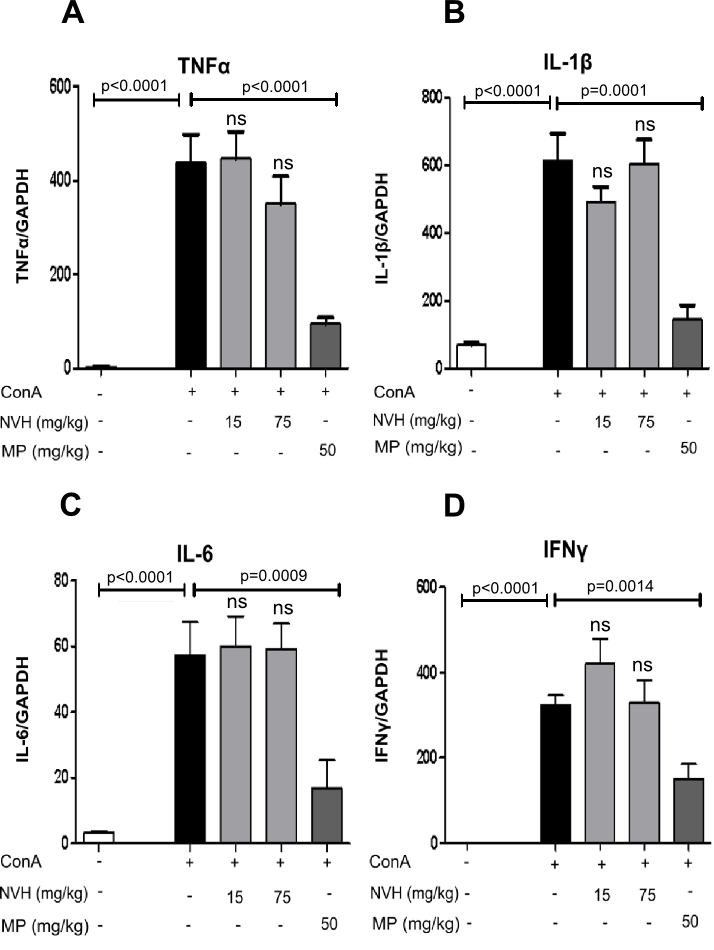

Supplement: Supplementary file 1 — Figure S1. Pro-inflammatory cytokine expression in Concanavalin A-intoxicated mice. Mice were pretreated with the extract or methylprednisolone and acute liver injury was induced with Concanavalin A intravenous injection after the last treatment. Bar graphs show Tumor Necrosis Factor alpha (A), Interleukin-1 beta (B), Interleukin-6 (C) and Interferon gamma (D) liver expression. Data are expressed as mean ± Standard Error of Mean. Significant Dunn’s post tests are indicated as *p < 0.05; **p < 0.01; ***p < 0.001; ns: non-significant. The p-value indicates the Mann-Whitney test. Two independent experiments; n ≥ 10 in each group. TNFα: Tumor Necrosis Factor alpha; IL-1β: Interleukin-1 beta; IL-6: Interleukin-6; IFNγ: Interferon gamma; NVH: Neoboutonia velutina aqueous extract; MP: Methylprednisolone; ConA: Concanavalin A; GAPDH: Glyceraldehyde-3-Phosphate Dehydrogenase. (TIFF 2650 kb) [file 12906_2018_2091_MOESM1_ESM.tif]

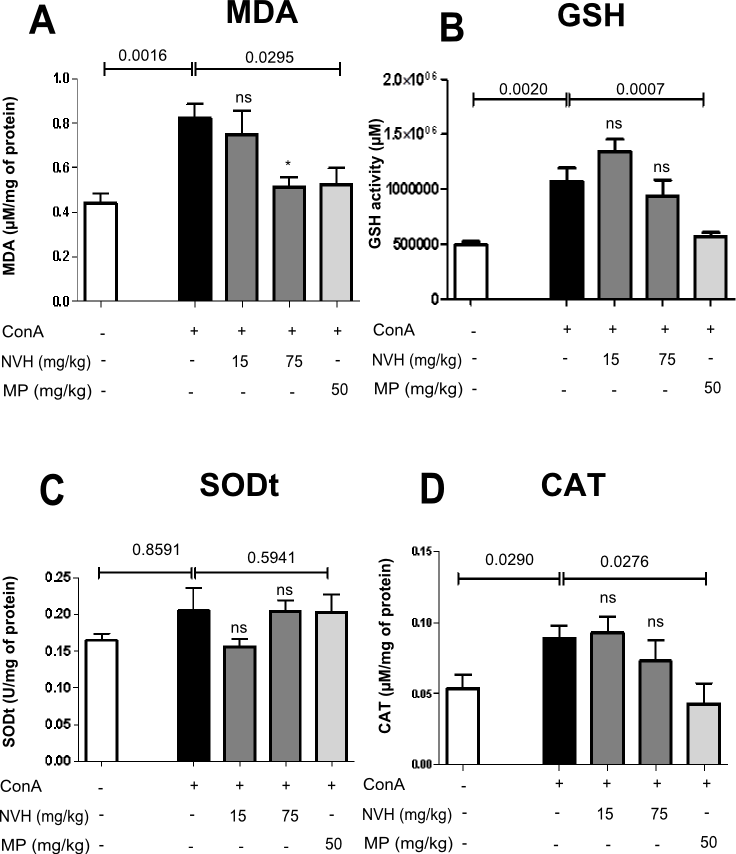

Supplement: Supplementary file 2 — Figure S2. Lipid peroxidation product and endogen antioxidant activity in Concanavalin A-intoxicated mice. Mice were pretreated with the extract or methylprednisolone and acute liver injury was induced with Concanavalin A intravenous injection after the last treatment. Bar graphs show Malondialdehyde level (A), Glutathione (B), total Superoxide dismutase (C) and Catalase (D) activity in mice. Data are expressed as mean ± Standard Error of the Mean. Significant Dunn’s post tests are indicated as *p < 0.05; **p < 0.01; ***p < 0.001; ns: non-significant. The p-value indicates the Mann-Whitney test. Two independent experiments; n ≥ 10 in each group. MDA: Malondialdehyde, GSH: Glutathione; SOD: Superoxide dismutase; CAT: Catalase; NVH: Neoboutonia velutina aqueous extract; MP: Methylprednisolone (TIFF 1841 kb) [file 12906_2018_2091_MOESM2_ESM.tif]

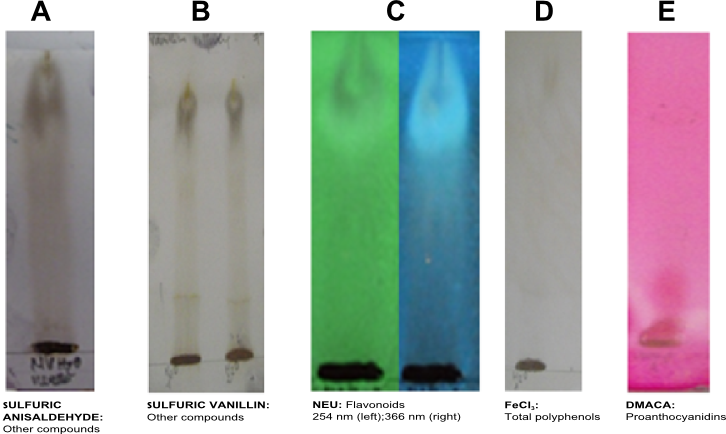

Supplement: Supplementary file 3 — Figure S3. NVH TLC for phytochemical analysis. Twenty μl of NVH (50 mg/mL) were deposited on a silica plate which was eluted using water: methanol: acetic acid (12.5:12.5:1). The eluted plate was dried before being soaked for 5 s in the appropriate reagent. A: sulfuric anisaldehyde reagent; B: sulfuric vanillin reagent; C: NEU reagent; D: FeCl3 reagent; E: Dimethylamino-cinnamaldehyde (DMACA) reagent. (TIFF 923 kb) [file 12906_2018_2091_MOESM3_ESM.tif]
